# A primary hydatid cyst in the mesorectum uncommon location – A rare case report

**DOI:** 10.1016/j.ijscr.2023.109061

**Published:** 2023-11-22

**Authors:** Murad Ibrahim, Afnan W.M. Jobran, Afnan Attalah, Ibrahim Abassi, Mohammad Baker Abu Isneineh

**Affiliations:** a Department of Microbiology and Immunology, Faculty of Medicine, AlQuds University, Jerusalem, Palestine; b Faculty of Medicine, AlQuds University, Jerusalem, Palestine; c Department of Physiology and Pharmacology, Faculty of Medicine, AlQuds University, Jerusalem, Palestine; d Department of Anatomy and Embryology, Faculty of Medicine, AlQuds University, Jerusalem, Palestine

**Keywords:** Mesorectum, Hydatid cyst, Unusual localization, Surgery, Echinococcosis

## Abstract

**Introduction and importance:**

The tapeworm *Echinococcus granulosus* sensu lato is the causative agent of cystic echinococcosis (CE), often known as hydatid disease. Over two-thirds of all occurrences of this zoonotic disease process in humans are caused by hepatic infection. Clinicians should have a low threshold to consider CE as a differential diagnosis in patients with positive serology and suggestive radiological findings, especially in endemic regions, because signs and symptoms are typically non-specific, especially in early disease.

**Case presentation:**

This is a case report of a 26-year-old male who presented with increasing lower abdominal discomfort, mild pain, sense of fullness in the lower abdomen, described as (I'm having a ball in my abdomen), with a history of early satiation and tenesmus, frequency of urine, and history of weight loss and general weakness of 10-months duration. The diagnosis of a hydatid cyst in the mesorectum was made. The cyst was completely excised via open surgery. No local recurrence has been detected up to the present time.

**Clinical discussion:**

Given how uncommon a site like this is, this case report helps broaden the differential diagnosis of soft tissue masses in such settings, especially in endemic areas. It also describes in great detail how these locations are affected by the hydatid disease.

**Conclusion:**

The mesorectal hydatid cyst was challenging to diagnose initially due to its infrequent incidence and uncommon location. In a few rare cases, the diagnosis of a hydatid cyst might be guided by the detection of the cyst membrane and daughter cysts in the germinal membrane.

## Highlights

• The mesorectal hydatid cyst was challenging to diagnose
• Its infrequent incidence and uncommon location.
• In a few rare cases, the diagnosis of a hydatid cyst might be guided by the detection of the cyst membrane and daughter cysts in the germinal membrane.

## 1 Introduction

The metacestode larval cystic stage of the tapeworm *Echinococcus granulosus* causes hydatid disease. Canids, which harbor the tapeworm, contract it when they consume the viscera of sheep that have viable hydatid cysts. In South and Central America, the Middle East, a few sub-Saharan African nations, and China, the disease is considered a serious public health issue. The majority of infections that are spread to intermediate hosts including people, sheep, and cattle in the Mediterranean region come from stray dogs and less from dogs that are kept as pets [1]. Seventy percent of hydatid cyst cases develop in the liver. A small percentage of the produced oncospheres' larval stage passes through the liver and are trapped in the pulmonary capillary bed; the oncospheres that escape the pulmonary capillary bed enter the systemic circulation and develop cysts in the lung, spleen, brain, or bones [1]. The infection's natural course varies. While some cysts spontaneously shrink, vanish, or calcify, others grow slowly, displacing or compressing good tissue and organs, and occasionally becoming problematic. The cyst typically grows between one and three centimeters in diameter per year [1].

In this case report, a hydatid cyst was found in the mesorectum, which is unusual. This case report contributes to diversifying the differential diagnosis of soft tissue masses in such a place, particularly in endemic areas, because such a site is exceedingly unusual. It also goes into great detail about how the hydatid illness manifests itself in these places.

## 2 Case report

26-year-old male works as a farmer with a free past medical history, Presented to surgery clinic with a history of abdominal pain during the last 10 months duration. His pain started as a mild lower abdominal discomfort without GIT or urinary symptoms 10 months ago.

Recently in the last few weeks, the patient had increasing lower abdominal discomfort, mild pain, sense of fullness in the lower abdomen, described as (I'm having a ball in my abdomen), with a history of early satiation and tenesmus, frequency of urine, and history of weight loss and general weakness.

**Fig. 1:**
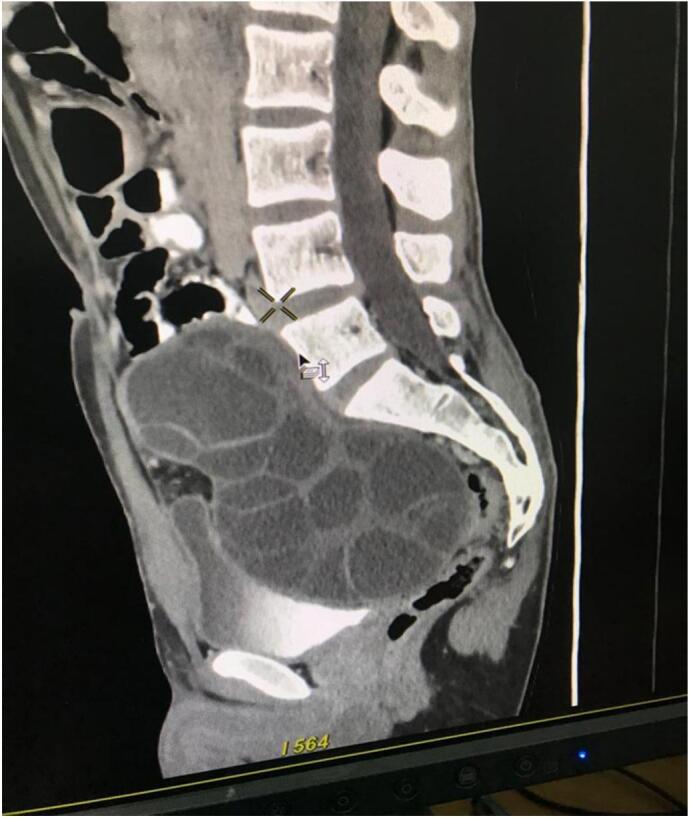
Abdomen CT scan.

Abdominal examination revealed a distended lower abdomen, a sense of deep-seated mass on palpation with mild tenderness, and no muscular guarding or rigidity. Ultrasonography (USG) abdomen revealed a multiseptated cyst (15 cm × 10 cm) filling the central area of the pelvis and extending into the abdominal cavity suggestive of a hydatid cyst, no other cyst was revealed in the abdomen. An abdominal CT scan was done, which showed: a huge lobulated cystic lesion with multiple septations occupying the pelvis and reaching the lower part of the abdominal cavity (Fig. 1).

**Fig. 2:**
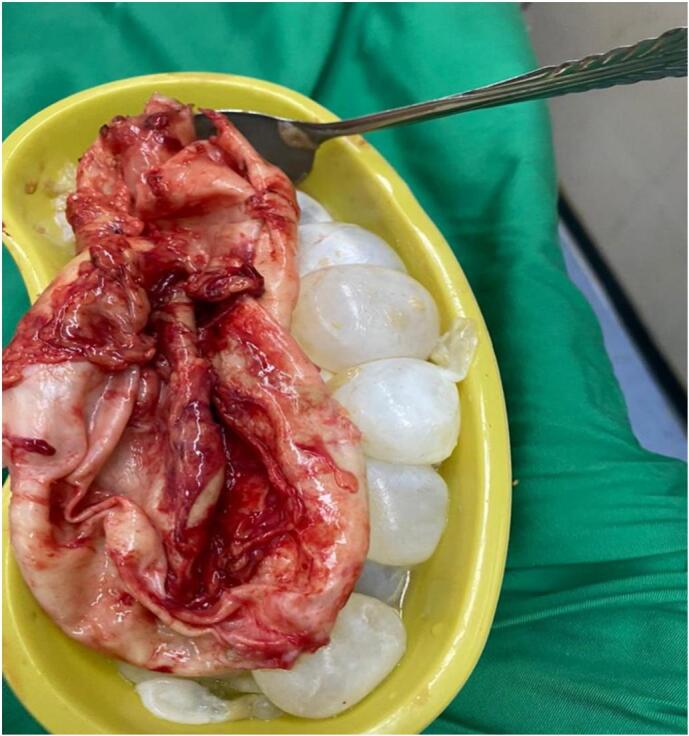
Evacuated cysts.

The patient underwent surgery, Through a midline incision the abdomen was opened in layers formal exploration was done as possible the cyst was found deeply seated in the pelvis adherent to the upper portion of the rectum and mesocolon inferiorly, compressing on the posterior wall of the urinary bladder anteriorly, and partially adherent to the small bowel loops and omentum. The cyst has its major adherent area to the medial side of the upper part of the rectum and mesorectum with small feeding blood vessels originating from the medial side of the mesorectum, five abdominal pads soaked with hypertonic saline were placed around the cyst. The feeding blood vessels were ligated and were cut. The cyst was completely excised after its content was evacuated. Two abdominal drains were inserted in the pelvis and the abdomen was closed in layers (Fig. 2).

Smooth postoperative course and the Patient was discharged albendazole was given for 4 weeks and the liver function test was checked. No findings associated with local or systemic hydatid disease were detected during the follow-up period.

**Fig. 3:**
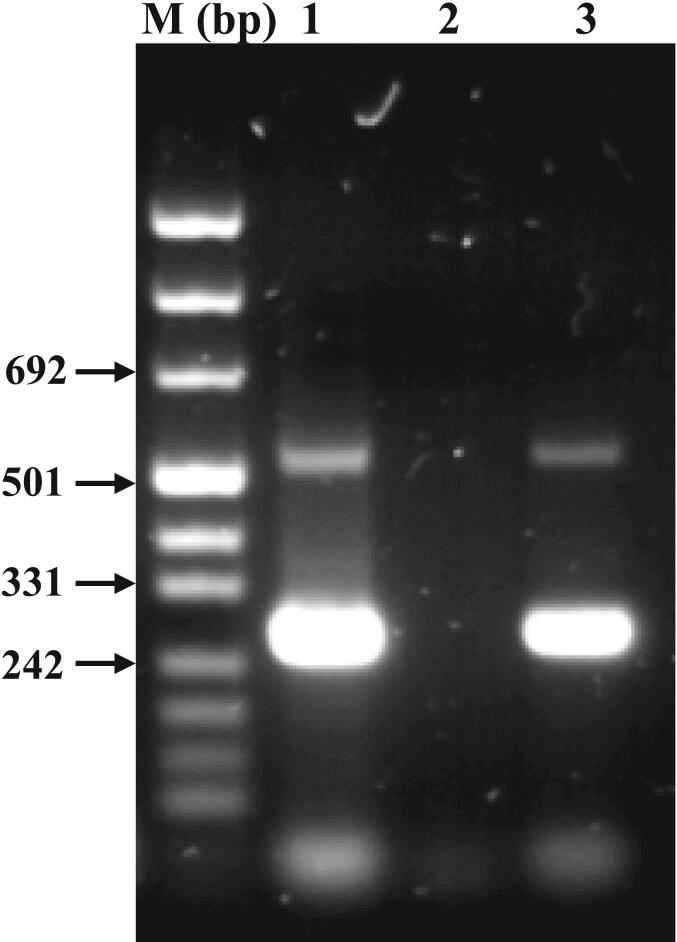
Agarose gel electrophoresis showing specific *E. granulosus* repeated sequence amplification from the patient's hydatid cyst inner membrane (1), negative control (2), and from *E. granulosus* genmic DNA (3). M: DNA size marker.

*E. granulosus* specific DNA amplification test: DNA was extracted from the isolated hydatid cyst, and then *E. granulosus* specific DNA fragments were amplified using a polymerase chain reaction system that targeted (EgG1 *Hae* III) repeated sequence (3). For this purpose, 0.3 g of the inner cyst membrane tissue was extracted using Qiagen *DNeasy* Blood and Tissue *Kit* (Qiagen, Germany), and the specific repeated sequence was amplified as it was previously described (3). Fig. 3 depicts specific *E. granulosus* amplified DNA repeat bands (about 270 bp and 540 bp) from both the isolated cyst DNA as well as from 0.1 ng of *E. granulosus* genomic DNA.

## 3 Discussion

Hydatidosis is a serious and chronic zoonotic disease in humans that is caused by dog tapeworm *Echinococcus granulosus*. Canids like dogs, wolves, and foxes serve as the primary hosts for *E. granulosus*, while sheep, goats, and cattle serve as intermediate hosts, Human beings is considered as coincidental intermediate host. The disease is more prevalent in the Middle East, Central Europe, Australia, and South America. The disease was not widespread among Somalis, according to research done to determine the frequency of hydatid disease in nomadic pastoralists residing in eastern Africa [2]. The case under discussion originates from Somaliland, an East African region situated in Hargeisa. Dog ownership is uncommon in the area, although keeping sheep is relatively widespread.

Any organ, including the kidneys, spleen, bile ducts, mesentery, brain, and soft tissue, can develop a hydatid cyst [3]. Intestinal tissue is where larvae develop from the eggs; the majority of the parasite oncospheres larval stage are stuck in the liver after invasion of the blood vessels. The larvae typically travel through the portal system to the liver, but occasionally they can get beyond the liver barrier and travel to the lungs and other internal organs, where they develop into tiny cysts [4]. For instances with single cysts in atypical locations, systemic diffusion via the lymphatic pathway is quite likely the passage route [5]. The larvae can also migrate through the intestinal wall and the venous mesenteric lymph arteries before settling in tissues and/or different intraabdominal organs [6]. Direct transmission from nearby sites may be another method of infection if a microrupture is present [5]. The absence of hepatic, pulmonary, or neighboring organ HD in our instance leads us to believe that transmural migration through the intestinal wall was preferable.

Preoperative diagnosis of a hydatid cyst can be challenging, as it may occasionally be mistaken for an abscess [7]. The most frequent clinical or exploratory finding of hydatid illness affecting soft tissues is a palpable lump [8]. Our patient complained of lower stomach discomfort, minor pain, and a feeling of satiety that was explained by the mass effect.

Hepatic hydatid cysts may be diagnosed by serological methods and using a variety of imaging modalities. Plain X-rays, ultrasounds, and CT scans were among the imaging procedures [9,10]. A spherical calcified lesion in the film may be visible on a plain X-ray [9]. The specific location and anatomical characteristics of the cyst will be revealed by an ultrasound scan and a CT scan [11]. The cyst has been sterilized, the danger of allergy has decreased, the tension in the cyst wall has decreased (thus lowering the risk of leakage during surgery), and the recurrence rate has decreased postoperatively [12]. For the treatment of hepatic hydatidosis, albendazole is much more effective than mebendazole, while benzimidazole treatment alone requires extended dosing over several weeks, with uncertain results in terms of response rates in individuals [13]. According to several studies, these medicines can provide a subjective improvement by killing the scolices and reducing hydatid fluid output, which causes the cyst to contract [14]. Within a few months to a few years, the cyst is replaced by a new granulation of tissue (regeneration) [15].

After a suitable period, operational spilling of cyst contents may result in local hydatid cyst recurrence [16]. The most often used form of treatment is complete excision of the germinal layer after injection of sporicidal drugs, which stops the germinal cyst from spreading to the surrounding tissue. One of the recommended sporicidal agents is hydrogen peroxide [17]. Up to 30 % of patients who get albendazole treatment for *E. granulosus* infections appear to be cured. Both the length of treatment and the dosage are crucial, and with albendazole, efficacy appears to rise with exposure up to three months in the more prevalent cyst sites. The debate over cyclical vs. continuous treatment is still open [18]. Our patient was only given albendazole for two weeks because his hydatid cyst was in a very uncommon location and there was no intraoperative leaking. Since he has been receiving follow-up care for the previous six months, no recurrence has been identified.

The rarity of this case made preoperative diagnosis difficult because the location of the cyst was very unusual. Intraoperative diagnosis of the typical germinal cyst membrane, with complete excision, was effective for hydatid cysts in this area. The work has been reported in line with the SCARE criteria [19].

## 4 Conclusion

To summarize, hydatid cysts should be considered in the differential diagnosis of pelvic masses, particularly in patients who have lived in endemic areas. To rule out alternative possibilities, proper imaging is essential. The treatment of choice is complete excision.

## Ethical approval

(Ethical Committee N° NAC 207) was provided by the Ethical Committee NAC of Alquds University, Jerusalem, Palestine on 12 July 2023.

## Source of funding

The study did not receive any funding.

## CRediT authorship contribution statement

Study guarantor; Mohammad Baker Abu Isneineh.

Data collection; Mohammad Baker Abu Isneineh and Ibrahim Abassi.

Writing the paper; Murad Ibrahim, Afnan Attalah, Ibrahim Abassi and Afnan W.M. Jobran.

Review and editing; Afnan W.M. Jobran.

## Guarantor

Mohammad Baker Abu Isneineh.

## Registration of research studies

Not applicable.

## Consent

Written informed consent was obtained from the patient's family for reporting this case and its associated images. The consent is available for review on request.

## Declaration of competing interest

There is no conflict of interest to declare.
